# Supplementary material for: The concentration of single-stranded DNA-binding proteins is a critical factor in recombinase polymerase amplification (RPA), as revealed by insights from an open-source system
Source: PeerJ. 2025 Aug 13;13:e19758. doi: 10.7717/peerj.19758 (PMC12357552; doi:10.7717/peerj.19758)
Supplement: Supplemental Information 1 [file peerj-13-19758-s001.docx]

**Table S1 RPA system using minimal components and easy purification**

| Enzymatic function | Protein name | MWM | Assembly | Source | Beneficial His-Tag Removal | Cloning |
| --- | --- | --- | --- | --- | --- | --- |
| SSB | gp32 | 33.5 kDa | dimer | This work. Synthetic gene  Plasmid 236044  Addgene | NO | pET19b-pss |
| Mediator | UvsY | 16 kDa | Heptamer (Gajewski et al. 2016) | This work. Synthetic gene  Plasmid 236045  Addgene | NO | pET19b-pps |
| Recombinase | UvsX | 44 kDa | filament | This work. Synthetic gene  Plasmid 236046 Addgene | YES (PreScission Protease) | pET19b-pps |
| Moloney murine leukemia virus (MMLV) Reverse transcriptase (RT)  Heat Resistance* | MMLV RT-4M | 75 kDa | Monomer | (Okano et al. 2017; Yasukawa et al. 2010)* | NO | pET-22b(+) |
| DNA polymerase | KF-BstDNApolI | 80 kDa | Monomer | This work courtesy of Dr. César Cardona. Synthetic gene  Plasmid #236047 Addgene | NO | pCOLDI |
| DNA polymerase | KF-BsuDNApolI | 80 kDa | Monomer | (Patchsung et al. 2023)  Plasmid # 163911 Addgene | NO | pET28b |
| APE1 nuclease | *Thermus thermophilus* HB8  Endo IV | 39 kDa | Monomer | This work. Synthetic gene  Plasmid # 236048 Addgene | NO | pET19pps |
| Creatine Kinase | Muscle-type Chicken creatine kinase | 82 kDa | Dimer | (Shimizu et al. 2001) Plasmid #124134-Addgene | NO | pQE30-His-CKM |

* An alternative Moloney murine leukemia virus (MMLV) Reverse transcriptase (RT) dubbed pOpen-MMLV_RT (lacking Ribonuclease H activity) is available from Addgene courtesy of Drs. Drew Endy & Jennifer Molloy & FreeGenes Project (Addgene plasmid # 165556 ; http://n2t.net/addgene:165556 ; RRID:Addgene_165556)

**Table S2 Composition of the protein for storage and enzymatic assays.**

| Enzyme | Final storage buffer for snap freezing | | Final concentration for storage | Molar concentration for storage (as a monomer) | | Molar concentration during enzymatic Reactions (as a monomer) |
| --- | --- | --- | --- | --- | --- | --- |
| gp32 | 50 mM Tris HCl pH 7.5, 250 mM NaCl, 2 mM DTT, 0.2 mM EDTA, 10% glycerol | | 6 mg/ml | 149 µM | | 27 µM |
| UvsY | 50 mM Tris HCl pH 7.5, 250 mM NaCl, 2 mM DTT, 0.2 mM EDTA, 10% glycerol | | 2 mg/ml | 125 µM | | 1.8 µM |
| UvsX | 50 mM Tris HCl pH 7.5, 250 mM NaCl, 2 mM DTT, 0.2 mM EDTA, 10% glycerol | | 44 kDa 2 mg/ml | 45 µM | | 2.7 µM |
| heat-resistant Moloney murine leukemia virusRT | 50 mM Tris-HCl pH 7.5, 150 mM NaCl, 1 mM DTT, 0.1 mM EDTA, 10% Glycerol, 0.05% NP-40 | | 5 mg/ml | 66 µM | | 1 μM |
| KF-BstDNApolI | 10 mM Tris-HCl pH 7.1, 50 mM KCl, 2 mM DTT, 0.1 mM EDTA, 10% Glycerol, 0.1% Triton® X-100 | | 5 mg/ml | 62 µM | | 0.62 μM |
| KF-BsuDNApolI | 10 mM Tris-HCl pH 7.1, 50 mM KCl, 2 mM DTT, 0.1 mM EDTA, 10% Glycerol, 0.1% Triton® X-100 | | 5 mg/ml | 62 µM | | 0.62 μM |
| APE1(*Thermus Thermophilus*) | 50 mM Tris HCl pH 7.5, 200 mM NaCl, 10 % glycerol, 2 mM BME, and 2 mM EDTA | | 2 mg/ml | 51 µM | | 0.5 μM |
| Muscle-type Chicken creatine kinase | 20 mM Tris pH 8.0, 50 mM NaCl, 0.2 mM EDTA, 2 mM DTT 10% glycerol | 5 mg/ml | | | 61 µM | 1.2 μM |

**Table S3 Concentrations of T4 Homologous recombination proteins used for RPA reactions**

|  | Kojima *et al.*, 2021 | Molar concentration | Piepenburg *et al.*, 2006 | Molar concentration | Juma *et al.*, 2022; | Molar concentration | Basic RPA Freeze Dried Reaction Pellet  US9057097B2  United States | Molar concentration |
| --- | --- | --- | --- | --- | --- | --- | --- | --- |
| Uvs Y | 480 ng/μL | 30 μM | 30 ng/μl | 1.87 μM | 40 ng/µL | 2.5 μM | 88 ng/µL | 5.5 μM |
| Uvs X | 570 ng/μL | 12.7 μM | 120 ng/μl | 2.72 μM | 400 ng/µL | 9.09 μM | 260 ng/µL | 5.91 μM |
| gp32 | 340 ng/μL | 4.63 μM | 900 ng/μl | 26.8 | 400 ng/µL | 11.9 μM | 254 ng/µL | 7.58 μM |

**Table S4 Melting temperatures for T4-HR proteins and associated DNA polymerases**

|  | Reported Tm(°C) | Reference | Calculated Tm (°C) |
| --- | --- | --- | --- |
| UvsX | --- |  | 49 ± |
| UvsY | --- |  | 60 ± |
| gp32 | 50 | (Pant et al. 2004). | 49 ± |
| Bsu DNAP |  |  | 54 ± |
| Bst DNAP | 65 | (Kiefer et al. 1998) | 72 ± |
