# Supplementary material for: The concentration of single-stranded DNA-binding proteins is a critical factor in recombinase polymerase amplification (RPA), as revealed by insights from an open-source system
Source: PeerJ. 2025 Aug 13;13:e19758. doi: 10.7717/peerj.19758 (PMC12357552; doi:10.7717/peerj.19758)
Supplement: Supplemental Information 2 [file peerj-13-19758-s002.docx]

**SUPPLEMENTARY SYNTHETIC GENES AND PURIFICATION PROTOCOLS**

1. **Amino acid and nucleotide sequences of synthetic genes reported in this study**

**1.- T4 UvsX**

**a) Protein sequence**

MSDLKSRLIKASTSKLTAELTASKFFNEKDVVRTKIPMMNIALSGEITGGMQSGLLILAGPSKSFKSNFGLTMVSSYMRQYPDAVCLFYDSEFGITPAYLRSMGVDPERVIHTPVQSLEQLRIDMVNQLDAIERGEKVVVFIDSLGNLASKKETEDALNEKVVSDMTRAKTMKSLFRIVTPYFSTKNIPCIAINHTYETQEMFSKTVMGGGTGPMYSADTVFIIGKRQIKDGSDLQGYQFVLNVEKSRTVKEKSKFFIDVKFDGGIDPYSGLLDMALELGFVVKPKNGWYAREFLDEETGEMIREEKSWRAKDTNCTTFWGPLFKHQPFRDAIKRAYQLGAIDSNEIVEAEVDELINSKVEKFKSPESKSKSAADLETDLEQLSDMEEFNE*

**b) Synthetic gene (1188 nts)**

**CATATG**ATGAGCGATCTGAAGTCTCGGCTGATTAAAGCTAGTACTTCCAAACTCACTGCAGAACTTACCGCGAGTAAATTTTTTAATGAAAAGGATGTCGTCCGGACCAAAATTCCCATGATGAACATCGCATTGAGTGGCGAGATTACCGGTGGTATGCAGTCTGGTCTGCTGATCCTGGCGGGCCCCTCGAAGTCTTTCAAAAGTAACTTTGGCCTGACCATGGTCTCCTCTTATATGCGTCAGTATCCGGATGCTGTGTGCCTGTTCTACGATAGTGAATTTGGCATCACACCGGCATATTTACGTAGCATGGGCGTTGATCCGGAGCGCGTGATTCATACACCTGTGCAGAGCCTTGAACAATTGCGCATTGATATGGTGAATCAACTGGATGCCATCGAACGCGGGGAAAAAGTGGTGGTGTTCATCGACTCCCTGGGGAATCTGGCCTCAAAAAAAGAAACAGAGGACGCATTGAACGAGAAAGTTGTTTCTGACATGACGCGGGCCAAAACAATGAAGTCGTTATTTCGCATTGTGACCCCCTACTTCTCCACGAAAAATATTCCGTGTATTGCCATTAACCATACGTACGAGACTCAAGAAATGTTTAGCAAAACCGTCATGGGCGGAGGGACGGGACCGATGTATAGTGCGGATACGGTGTTTATCATTGGCAAACGCCAGATCAAAGATGGCAGTGACCTGCAAGGCTACCAGTTCGTGTTAAACGTAGAAAAAAGCCGCACCGTCAAAGAAAAGAGCAAGTTTTTTATCGATGTGAAATTCGACGGTGGTATTGATCCGTATAGCGGGTTATTGGATATGGCCCTGGAGCTGGGCTTTGTCGTTAAACCGAAAAACGGCTGGTATGCACGCGAATTTCTGGACGAAGAAACCGGGGAAATGATCCGTGAGGAAAAAAGCTGGCGCGCCAAGGATACCAATTGCACTACTTTCTGGGGCCCGCTGTTCAAACATCAGCCCTTTCGTGATGCGATTAAACGGGCCTACCAACTGGGTGCAATTGATAGCAATGAAATTGTGGAGGCAGAGGTGGATGAACTGATTAACTCAAAAGTGGAGAAATTCAAATCGCCGGAATCCAAATCAAAATCAGCAGCCGATCTGGAAACGGATCTGGAACAACTTAGCGATATGGAAGAGTTCAATGAA**TAAGGATCC**

**2.- T4 UvsY Mediator**

**a) Protein sequence**

MRLEDLQEELKKDVFIDSTKLQYEAANNVMLYSKWLNKHSSIKKEMLRIEAQKKVALKARLDYYSGRGDGDEFSMDRYEKSEMKTVLSADKDVLKVDTSLQYWGILLDFCSGALDAIKSRGFAIKHIQDMRAFEAGK*

**b) Synthetic gene (426 nts)**

CATATGATGCGTCTGGAAGACCTGCAGGAAGAACTGAAAAAAGATGTGTTCATTGATTCCACGAAACTGCAATATGAAGCGGCTAACAACGTGATGCTGTATAGCAAATGGCTGAACAAACACAGCAGCATTAAAAAGGAAATGCTGCGCATCGAAGCACAAAAAAAAGTGGCATTAAAGGCACGTCTGGATTACTACTCGGGACGCGGTGACGGCGATGAATTTAGCATGGACCGCTATGAGAAGTCTGAGATGAAGACGGTGTTATCTGCGGACAAAGACGTCCTGAAAGTCGATACCAGCCTTCAGTATTGGGGCATCCTGTTAGACTTTTGCTCAGGGGCTCTGGATGCGATCAAGAGCCGTGGCTTTGCCATTAAACATATTCAGGACATGCGGGCATTCGAAGCGGGCAAATGAGGATCC

**3.- T4 gp32 Single-Stranded Binding Protein**

**a) Protein sequence**

MFKRKSTAELAAQMAKLNGNKGFSSEDKGEWKLKLDNAGNGQAVIRFLPSKNDEQAPFAILVNHGFKKNGKWYIETCSSTHGDYDSCPVCQYISKNDLYNTDNKEYSLVKRKTSYWANILVVKDPAAPENEGKVFKYRFGKKIWDKINAMIAVDVEMGETPVDVTCPWEGANFVLKVKQVSGFSNYDESKFLNQSAIPNIDDESFQKELFEQMVDLSEMTSKDKFKSFEELNTKFGQVMGTAVMGGAAATAAKKADKVADDLDAFNVDDFNTKTEDDFMSSSSGSSSSADDTDLDDLLNDL*

**b) Synthetic gene (918 nts)**

CATATGATGTTCAAGCGGAAGAGCACAGCGGAACTGGCGGCGCAGATGGCGAAATTGAATGGTAACAAAGGTTTCTCCTCAGAAGATAAAGGTGAATGGAAACTCAAACTTGATAATGCGGGGAACGGGCAGGCCGTGATTCGTTTTCTGCCGAGTAAAAATGATGAACAAGCTCCGTTTGCCATCCTGGTGAATCACGGCTTTAAAAAAAATGGTAAATGGTACATTGAGACGTGCTCGTCTACTCATGGTGACTACGATTCCTGCCCGGTGTGCCAATATATCTCCAAAAATGACTTGTACAATACTGATAACAAAGAATATTCTCTGGTTAAACGCAAAACGTCTTACTGGGCGAACATTCTGGTGGTAAAGGACCCGGCCGCTCCGGAAAACGAAGGCAAAGTTTTTAAATACCGCTTCGGAAAAAAAATCTGGGATAAAATTAATGCAATGATTGCCGTGGACGTCGAGATGGGTGAAACCCCGGTTGATGTTACCTGTCCCTGGGAGGGCGCCAATTTTGTGTTGAAGGTTAAACAGGTAAGCGGATTTAGTAACTACGATGAATCAAAGTTCCTGAACCAGTCAGCTATCCCCAATATTGATGACGAATCCTTTCAGAAAGAACTCTTCGAACAGATGGTAGATCTTAGCGAAATGACATCTAAAGATAAATTCAAATCCTTTGAGGAACTGAATACCAAGTTCGGGCAGGTGATGGGGACGGCCGTAATGGGCGGCGCAGCGGCGACTGCGGCGAAAAAAGCAGATAAAGTAGCCGACGATCTGGATGCTTTCAATGTAGATGATTTTAACACCAAGACCGAGGATGATTTTATGTCCTCAAGCTCCGGTAGCTCGTCAAGTGCTGATGATACCGATCTTGATGATCTGCTGAATGATCTGTAAGGATCC

**4.- Endo IV from Thermus thermophilus**

**a) Protein sequence**

MPRYGFHLSIAGKKGVAGAVEEATALGLTAFQIFAKSPRSWRPRALSPAEVEAFRALREASGGLPAVIHASYLVNLGAEGELWEKSVASLADDLEKAALLGVEYVVVHPGSGRPERVKEGALKALRLAGVRSRPVLLVENTAGGGEKVGARFEELAWLVADTPLQVCLDTCHAYAAGYDVAEDPLGVLDALDRAVGLERVPVVHLNDSVGGLGSRVDHHAHLLQGKIGEGLKRVFLDPRLKDRVFILETPRGPEEDAWNLRVFRAWLEEA*

**b) Gene**

NC_006461.1:c800688-799876 *Thermus thermophilus* HB8, complete sequence

ATGCCGCGCTACGGGTTCCACCTTTCCATCGCCGGGAAAAAGGGCGTGGCCGGGGCGGTGGAGGAAGCCACCGCCCTCGGCCTCACCGCTTTCCAGATCTTCGCCAAAAGCCCGCGGAGCTGGCGCCCAAGGGCCCTCTCCCCGGCCGAGGTGGAGGCCTTCCGCGCCTTAAGGGAGGCCTCCGGGGGCCTCCCCGCCGTGATCCACGCCTCCTACCTGGTCAACCTGGGGGCGGAGGGGGAGCTTTGGGAGAAGAGCGTGGCGAGCCTGGCGGACGACCTGGAGAAGGCCGCCCTCCTCGGGGTGGAGTACGTGGTCGTCCACCCCGGCTCGGGCCGCCCCGAGCGGGTCAAGGAAGGGGCCCTCAAGGCCCTGCGCCTCGCCGGCGTCCGCTCCCGCCCCGTCCTCCTCGTGGAGAACACCGCTGGGGGTGGGGAGAAGGTGGGGGCGCGGTTTGAGGAGCTCGCCTGGCTCGTGGCGGACACCCCCCTCCAGGTCTGCCTGGACACCTGCCACGCCTACGCCGCCGGGTACGACGTGGCCGAGGACCCCTTGGGGGTCCTGGACGCCTTGGACCGGGCCGTGGGCCTGGAGCGGGTGCCCGTGGTCCACCTCAACGACTCCGTGGGCGGCCTCGGAAGCCGCGTGGACCACCACGCCCACCTCCTCCAGGGAAAGATCGGGGAGGGGCTCAAGCGCGTCTTTTTGGACCCGAGGCTCAAGGACCGGGTCTTCATCCTGGAAACCCCCAGGGGACCGGAGGAGGACGCCTGGAACCTCCGGGTCTTTAGGGCCTGGCTCGAGGAGGCCTAA

**SUPPLEMENTARY MATERIAL 2 PURIFICATION PROTOCOLS**

**Purification of UvsX and gp32**

The plasmid pET19b-(pps) containing UvsX or gp32 were transformed into the *E. coli* strain BL21 (DE3) / pKJE7 (Takara) that co-expresses chaperones. The transformed cells were grown in LB medium supplemented with chloramphenicol and ampicillin to an OD600 of 0.6-0.8. Reaching this point, it is induced with 0.5 mM Isopropyl-β-D-thiogalactoside (IPTG). The culture is left stirring at 16 ° C for 16 hrs. After the time has elapsed, the cells are collected by centrifugation at 6,000 rpm and 4 ° C for 10 min. The bacteria pellet was resuspended in lysis buffer (50 mM Tris HCl pH 7.5, 500 mM NaCl, 10 mM Imidazole, 10% glycerol). Once the pellet is resuspended, it is sonicated on ice and subsequently centrifuged for 30 min at 15,000 rpm and 4 °C. The supernatant is passed through a nickel column previously equilibrated with lysis buffer. Washes are carried out with 10-, 20-, and 40-mM imidazole in lysis buffer. The protein is eluted with 500 mM imidazole in lysis buffer. The elution fraction is dialyzed ON in dialysis buffer using a 10 kDa cutoff dialysis bag (50 mM Tris HCl pH 7.5, 200 mM NaCl, 2 mM BME, 1 mM EDTA, 10% glycerol. Dialysis was carried out in the presence of home-made PreScission protease to cleave its histidine tag (we typically add 0.1mg of highly purified PreScission protease per 2 liters of cell bacterial culture) and leave the protein at 4 ° C between 12 to 14 hours with moderate agitation. The protein is diluted 1: 4 in Buffer 0 (50 mM Tris HCl pH 7.5, 2 mM BME, 1 mM EDTA 10% glycerol) and is passed through a Q column. Elution is performed by saline gradient from 50 mM to 1M NaCl in Buffer 0. Protein fractions are concentrated to 2 mg/ml and dialyzed ON in storage buffer (50 mM Tris HCl pH 7.5, 250 mM NaCl, 2 mM DTT, 0.2 mM EDTA, 10% glycerol) using a dialysis bag of 10 kDa and subject to snap freezing

**Purification of UvsY**

The plasmid pET19-b(pps) UvsY was transformed into the E. coli strain BL21 (DE3) / pKJE7 that co-expresses chaperones (Takara). The transformed cells are grown in LB medium supplemented with ampicillin to an OD600 of 0.6-0.8. Reaching this point, it is induced with 0.5 mM Isopropyl-β-D-thiogalactoside (IPTG). The culture is left stirring at 16 ° C for 16 hrs. After the time has elapsed, the cells are collected by centrifugation at 6,000 rpm and 4 ° C for 10 min. The bacteria pellet is resuspended in lysis buffer (50 mM Tris HCl pH 7.5, 500 mM NaCl, 10 mM Imidazole, 10% glycerol). Once the pellet is resuspended, it is sonicated on ice and subsequently centrifuged for 30 min at 15,000 rpm and 4 ° C. The supernatant is passed through a nickel column previously equilibrated with lysis buffer. Washes are carried out with 10, 20, and 40 mM imidazole in lysis buffer. The protein is eluted with 500 mM imidazole in lysis buffer. The elution fraction is dialyzed ON in cutting buffer (50 mM Tris HCl pH 7.5, 250 mM NaCl, 2 mM DTT, 10% glycerol). The protein is diluted 1: 4 in Buffer 0 (50 mM Tris HCl pH 7.5, 2 mM DTT, 1 mM EDTA 10% glycerol) and is passed through a Q column. Elution is performed by saline gradient from 50 mM to 1M NaCl in Buffer 0. Proteins were concentrated to approximately 2 mg/ml and d dialyzed ON in storage buffer (50 mM Tris HCl pH 7.5, 250 mM NaCl, 2 mM DTT, 0.2 mM EDTA, 10% glycerol) using a dialysis bag of 10 kDa and subject to snap freezing

**Large Fragment of Bst and Bsu DNA polymerases**

This protocol relies on the protocol by the Chaput group with modifications [58]. A pCOLD-BstDNApol (AmpR) plasmid was transformed into strain BL21 (DE3)/pKJE7(Takara). The transformed cells were grown at 37 ° C and 200 rpm in LB medium with antibiotic until reaching an OD600 of 0.6. His Bst expression was induced with 1 mM isopropyl β-D-thiogalactoside (IPTG) at 16 ° C and 200 rpm for 16 hr. The cells were collected by centrifugation at 6,000 rpm and 4 ° C for 10 min. The resuspended pellet was sonicated in lysis buffer (50 mM Tris-HCl pH 7.5, 100 mM NaCl, 1 mM EDTA, 10 mM BME, 0.1% v / v NP-40 and 0.1% v / v Tween20). The lysate was centrifuged at 15,000 rpm and 4 ° C for 30 min. The supernatant was incubated for 20 min at 60 ° C and centrifuged at 15,000 rpm and 4 ° C for 30 min. The supernatant was dialyzed for 3 h in Buffer A (50 mM Tris-HCl pH 7.5, 100 mM NaCl) and loaded on a nickel column previously equilibrated with Buffer A. The column was washed with 10 and 20 mM imidazole in Buffer A. The protein was eluted with Buffer EB (50 mM Tris-HCl pH 7.5, 100 mM NaCl, 500 mM imidazole). The elution fraction was dialyzed ON in Buffer B (50 mM Tris-HCl pH 7.5, 20 mM KCl, 1 mM EDTA, 1 mM DTT). The dialyzed protein was loaded on a heparin column and eluted with a gradient from 20 to 1M in Buffer B. The fractions containing the Bst were dialyzed ON in a storage buffer (10 mM Tris-HCl pH 7.1, 50 mM KCl, 2 mM DTT, 0.1 mM EDTA, 10% Glycerol, 0.1% Triton® X-100) using a dialysis bag of 10 kDa and subject to snap freezing

**MMLV-RT 4M (mutante termoestable de MMLV-RT)**

This MMLVRT-4M is a quadruple mutant of wild-type MMLVRT. These mutations abolish RNase H activity and confers thermotolerance to 60 °C [44, 45]. The plasmid was transformed into strain BL21 (DE3) / pKJE7 that co-expresses chaperones (Takara). The transformed cells were grown at 37 °C and 200 rpm in LB medium with antibiotic until reaching an OD600 of 0.6. MMLV-RT 4M expression was induced with 1 mM isopropyl β-D-thiogalactoside (IPTG) at 16 ° C and 200 rpm for 16 hr. The cells were collected by centrifugation at 6,000 rpm and 4 ° C for 10 min. The resuspended pellet was sonicated in lysis buffer (50 mM Tris-HCl pH 7.5, 300 mM NaCl, 10 mM imidazole, 5% glycerol). The lysate was centrifuged at 15,000 rpm and 4 ° C for 30 min. The supernatant was loaded onto a nickel column previously equilibrated with lysis buffer. The column was washed with 10 and 20 mM of imidazole in lysis buffer. The protein was eluted with buffer EB (50 mM Tris-HCl pH 7.5, 300 mM NaCl, 500 mM imidazole, 5% glycerol). We add immediately after eluting: 2 mM EDTA and 5 mM DTT. The elution fraction was dialyzed in dialysis buffer (50 mM HEPES pH 7.5, 40 mM NaCl, 2 mM EDTA, 5 mM DTT, 5% glycerol). The protein was loaded onto a Mono-S column balanced with dialysis buffer. Elution was performed with a gradient from 40 to 1 M NaCl in dialysis buffer. The fractions with MMLRT-4M were dialyzed ON in a storage buffer (50 mM Tris-HCl pH 7.5, 150 mM NaCl, 1 mM DTT, 0.1 mM EDTA, 10% Glycerol, 0.05% NP-40) using a dialysis bag of 10 kDa and subject to snap freezing.

**Creatine Kinase**

The plasmid encoding Creatine kinase was used to transform a BL21 (DE3) / pKJE7 (Takara) that co-expresses chaperones cells were transformed with the plasmid and grown in LB medium until OD600 of 0.6. Induction was carried out with 1 mM IPTG at 16 °C for 16 h. Collect pellets by centrifugation at 6,000 rpm and 4 ºC for 10 min. The pellet was resuspended in B. Lysis (50 mM Tris-HCl pH 8, 200 mM NaCl, 20 mM imidazole), the lysate was by sonication. Centrifuge at 15,000 rpm for 30 min. The supernatant was loaded onto a 5 ml nickel column. Wash with 50 ml B. Lysis and Wash with 50 ml B. Lysis with 40 mM Imidazole. Elute with 15 ml of B. Lysis with 500 mM Imidazole. The elution fraction was dialyzed ON in B. Dial (50 mM Tris-HCl, 20 mM NaCl, 10 mM BME, 1 mM EDTA, pH 8.0). The dialyzed protein was loaded onto a 5 mL Fast Flow Q Sepharose column. Elution was performed with a gradient from 20 to 1 M NaCl in B. The fractions containing AK were mixed and dialyzed in storage buffer (20 mM Tris pH 8.0, 50 mM NaCl, 0.2 mM EDTA, 2 mM DTT 10% glycerol) using a dialysis bag of 10 kDa and subject to snap freezing.

**Purification protocol of Endo IV from *Thermus thermophilus***

The plasmid pET19b-(pps)*Thermus thermophilus* EndoIV was transformed into strain BL21 DE3/pKJE7 (Takara) (Cm). The transformed cells are grown in LB medium with the corresponding antibiotics (Amp and Cm), at 37°C and 200 rpm until reaching an OD of 0.6. Induction was carried out with 0.4 mM IPTG at 16°C and 200 rpm for 16 h. The pellet is collected by centrifugation at 6000 rpm and 4°C for 10 minutes. The pellet was resuspended in 40 ml of lysis buffer (50 mM Tris HCl pH 7.5, 100 mM NaCl, 10 mM imidazole, 10 % glycerol 2 mM BME) Once the pellet is resuspended, it is sonicated on ice and the lysate is subsequently clarified by centrifugation, 4°C, 30 min, 15,000 rpm. The supernatant is divided into 20 and 20 ml and heated at 75°C for 20 minutes. Incubate for 20 minutes on ice and centrifuge again, 30 minutes, 4°C, 15,000 rpm. The supernatant is loaded onto a 1 mL nickel column previously equilibrated with lysis buffer. The column is washed with 30 ml of Lysis buffer supplemented with 10, 20, and 30 mm of Imidazole. The purified Endo IV protein is eluted with 5 ml of 500 mM of Imidazole in lysis buffer. The eluted fraction was dialyzed overnight using a dialysis bag of 10 kDa and stored at -80 C in 50 mM Tris HCl pH 7.5, 200 mM NaCl, 10 % glycerol, 2 mM BME, and 2 mM EDTA by snap freezing.

**SUPPLEMENTARY MATERIAL 3 SARS- Cov2 genes cloned into pET28b**

To validate the RPA reaction, we decided to use a synthetic DNA coding for segments of the SARS COV-2 RNA polymerase gene (Red), E gene (light blue), N gene (orange) and the human RNAPase gene (as a positive control). This DNA segment in green was cloned between the *Nco* I and *BamH* I para sites of a vector pET28 (kanamycin resistance). Within the synthetic gene two T7 RNAP promoter sequences were added (colored in purple and before a restriction site Sal I)

CCATGGCAAGTATTGA**GTGAAATGGTCATGTGTGGCGG**TTCACTATATGTTAAACCAGGTGGAACCTCATCAGGAGATGCCACAACTGCT**TATGCTAATAGTGT**TTTTAACATTTGTCAAGCTGTCACGGCCAATGTTAATGCACTTTTATCTACTGATGGTAACAAAATTGCCGATAAGTATGTCCGCAATTTACAACACAGACTTTATGAGTGTCTCTATAGAAATAGAGATGTTGACACAGACTTTGTGAATGAGTTTTACGCATATTTGCGTAAACATTTCTCAATGATGATACTCTCTGACATGTACTCATTCGTTTCGGAAGAGACAG***GTACGTTAATAGTTAATAGCGT***ACTTCTTTTTCTTGCTTTCGTGGTATTCTTGCTAGTTACACTAGCCATCCTTACTGCGCTTCGAT**TGTGTGCGTACTGCTGCAATAT**TGTTAACGTGAGTCTTGTAAAACCTTCTTTTTACGTTTACTCTCGTGTTAAAAATCTGAATTCTTCTAGAGTTCCTGATCTTCTGGTCTAAACGAgtcgac**AAATTAATACGACTCACTATA**GGGAGACCTATGTCTGATAATG**GACCCCAAAATCAGCGAAAT**GCACCCCGCATTACGTTTGGTGGACCCT**CAGATTCAACTGGCAGTAACCAGA**ATGGAGAACGCAGTGGGGCGCGATCAAAACAACGTCGGCCCCAAGGTTTACCCAATAATACTGCGTCTTGGTTCACCGCTCTCACTCAACATGGCAAGGAAGACCTTAAATTCCCTCGAGGACAAGGCGTTCCAATTAACACCAATAGCAGTCCAGATGACCAAATTGGCTACTACCGAAGAGCTACCAGACGAATTCGTGGTGGTGACGGTAAAATGAAAGATCTCAGTCCAAGATGGTATTTCTACTACCTAGGAACTGGGCCAGAAGCTGGACTTCCCTATGGTGCTAACAAAGACGGCATCATATGGGTTGCAACTGAGGGAGCCTTGAATACACCAAAAGAT**CACATTGGCACCCGCAATC**CTGCTAACAATGCTGCAATCGTGCTACAACTTCCTCAAGGAACAACATTGCCAAAAGGCTTCTACGCAGAAGGGAGCAGAGGCGGCAGT**CAAGCCTCTTCTCGTTCCTC**ATCACGTAGTCGCAACAGTTCAAGAAATTCAACTCCAGGCAGCAGTAGGGGAACTTCTCCTGCTAGAATGGCTGGCAATGGCGGTGATGCTGCTCTTGCTTTGCTGCTGCTTGACAGATTGAACCAGCTTGAGAGCAAAATGTCTGGTAAAGGCCAACAACAACAAGGCCAAACTGTCACTAAGAAATCTGCTGCTGAGGCTTCTAAGAAGCCTCGGCAAAAACGTACTGCCACTAAAGCATACAATGTAACACAAGCTTTCGGCAGACGTGGTCCAGAACAAACCCAAGGAAATTTTGGGGACCAGGAACTAATCAGACAAGGAACTGA**TTACAAACATTGGCCGCAAA**TTGCACAATTTGCCCCCAGCGCTTCAGCG**TTCTTCGGAATGTCGCGC**ATTGGCATGGAAGTCACACCTTCGGGAACGTGGTTGACCTACACAGGTGCCATCAAATTGGATGACAAAGATCCAAATTTCAAAGATCAAGTCATTTTGCTGAATAAGCATATTGACGCATACAAAACATTCCCACCAACAGAGCCTAAAAAGGACAAAAAGAAGAAGGCTGATGAAACTCAAGCCTTACCGCAGAGACAGAAGAAACAGCAAACTGTGACTCTTCTTCCTGCTGCAGATTTGGATGATTTCTCCAAACAATTGCAACAATCCATGAGCAGTGCTGACTCAACTCAGGCCTAAgtcgac**AAATTAATACGACTCACTATA**GGGAGACCTGGACTTCAGCATGGCGGTGTTTGC**AGATTTGGACCTGCGAGC**GGGTTCTGACCTGAAGGCTCTGCGCGG**ACTTGTGGAGACAGCCGCTC**ACCGTGAGTTGCGGTCT gtcgacGGATCC
