# Supplementary figures and images for: The concentration of single-stranded DNA-binding proteins is a critical factor in recombinase polymerase amplification (RPA), as revealed by insights from an open-source system
Source: PeerJ. 2025 Aug 13;13:e19758. doi: 10.7717/peerj.19758 (PMC12357552; doi:10.7717/peerj.19758)

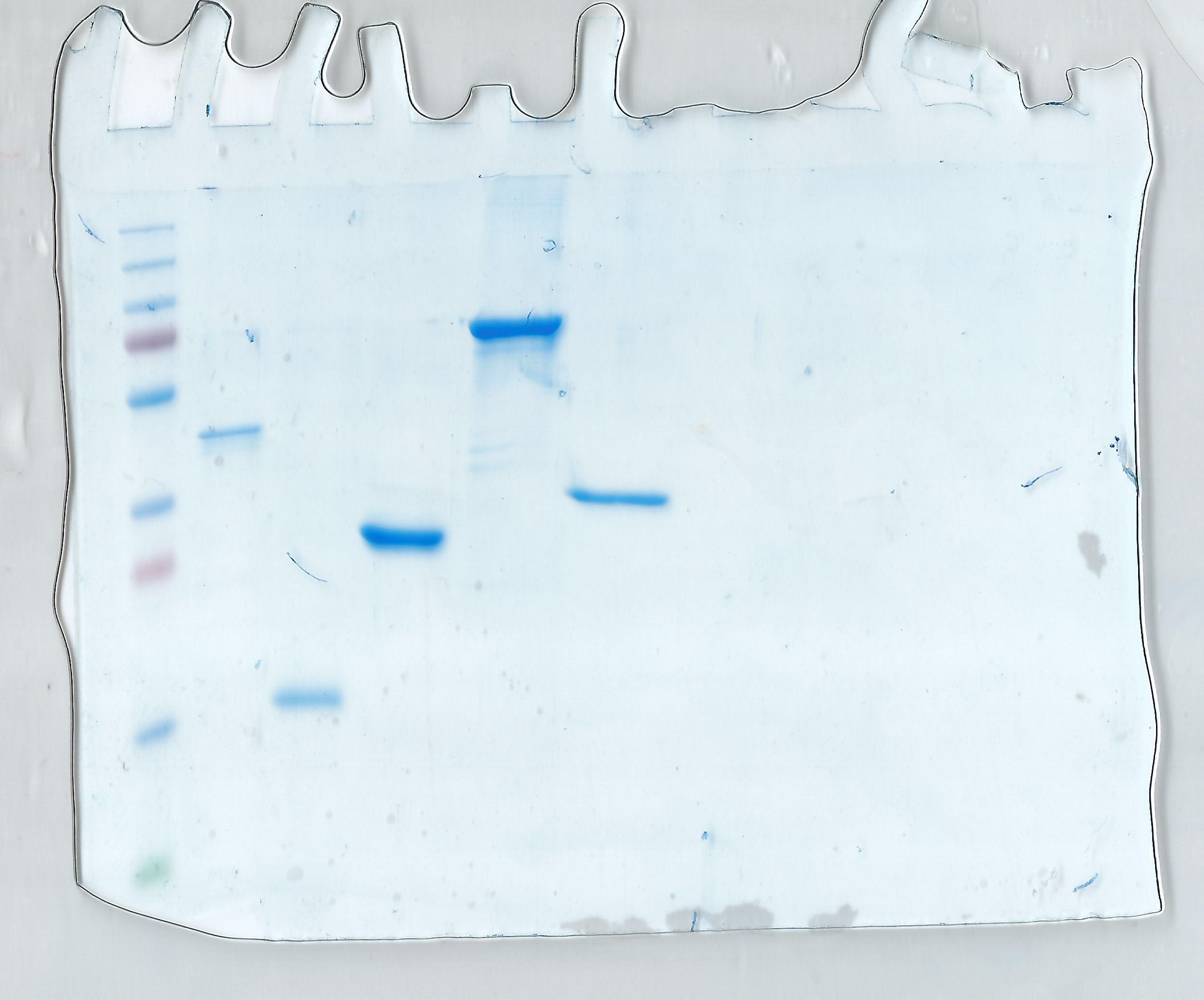

Supplement: Supplemental Information 3 [file peerj-13-19758-s003.zip › Fig1_2_3/Figure 1/Figure 1B/FIG1B.jpg]

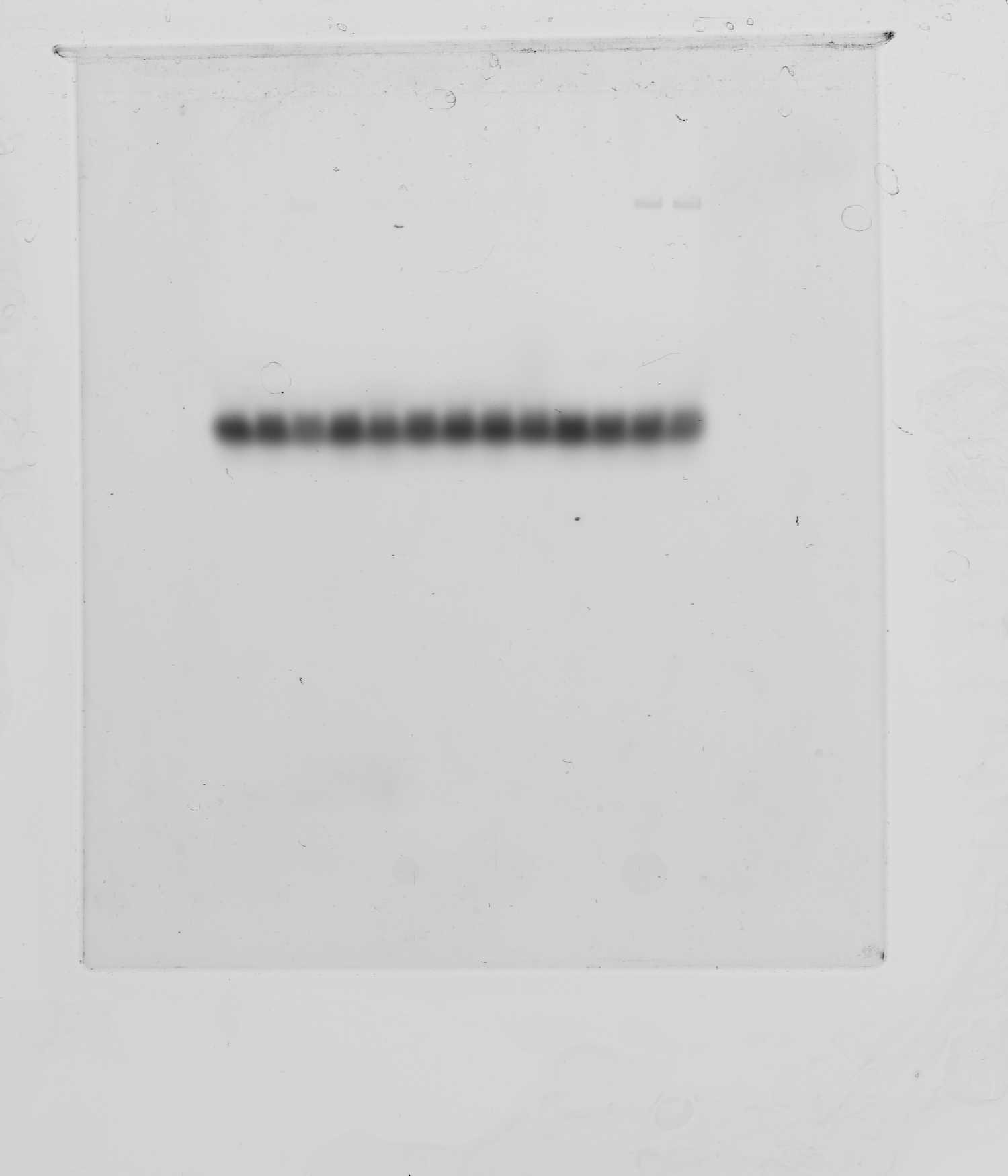

Supplement: Supplemental Information 3 [file peerj-13-19758-s003.zip › Fig1_2_3/Figure 1/Figure 1C/Figure1C.gel]

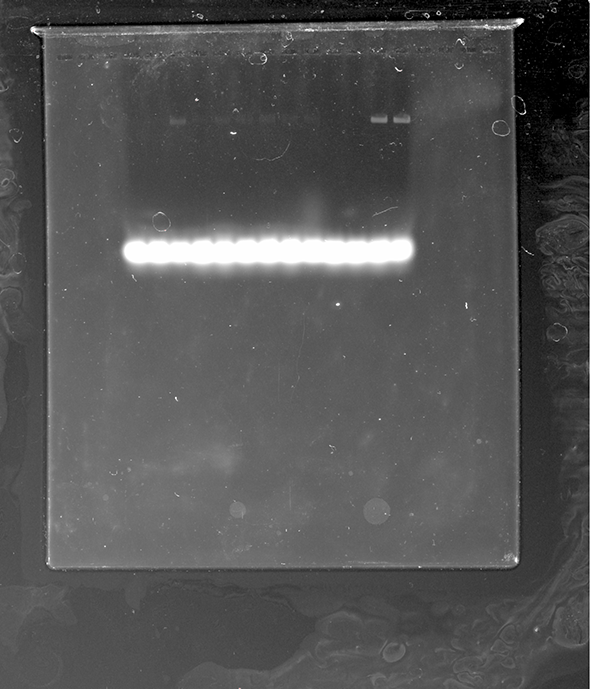

Supplement: Supplemental Information 3 [file peerj-13-19758-s003.zip › Fig1_2_3/Figure 1/Figure 1C/Figure1C.tif]

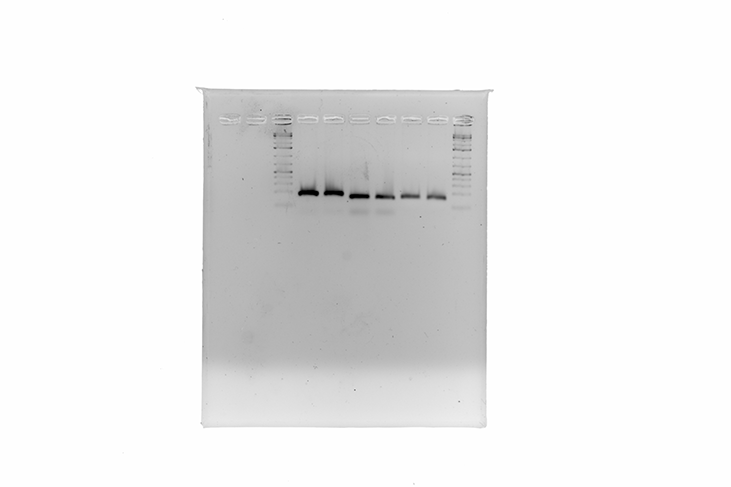

Supplement: Supplemental Information 3 [file peerj-13-19758-s003.zip › Fig1_2_3/Figure 3/Figure 3B/Fig3B.tif]

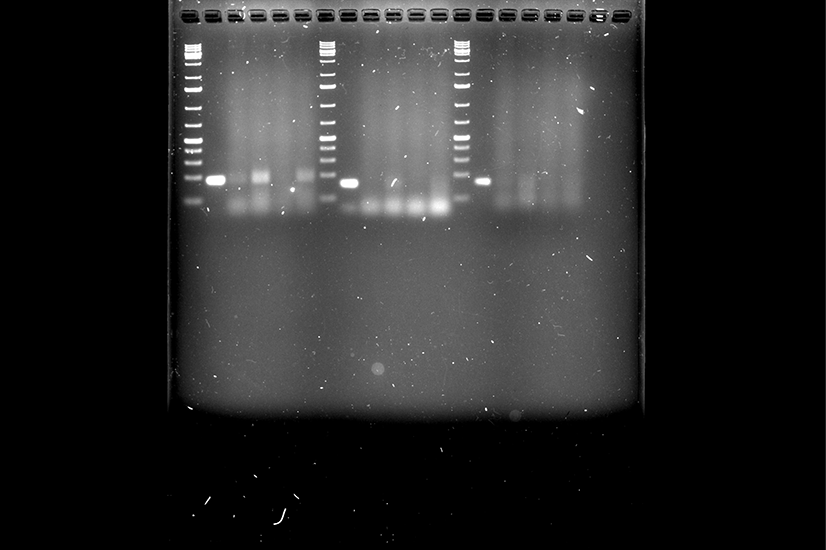

Supplement: Supplemental Information 3 [file peerj-13-19758-s003.zip › Fig1_2_3/Figure 3/Figure 3c/Fig3C.tif]

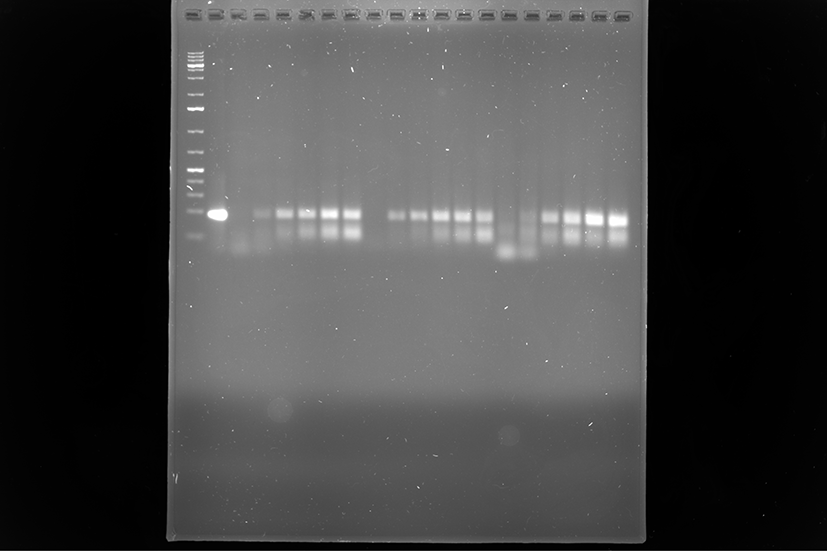

Supplement: Supplemental Information 3 [file peerj-13-19758-s003.zip › Fig1_2_3/Figure 3/Figure 3D/Fig3D.tif]

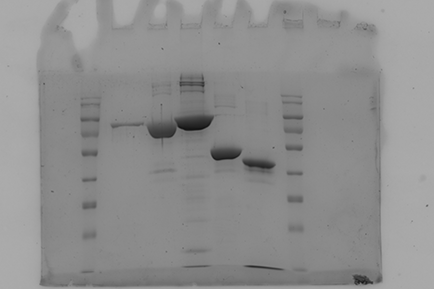

Supplement: Supplemental Information 3 [file peerj-13-19758-s003.zip › Fig1_2_3/Figure 2/Figure 2A/Fig2A.tif]

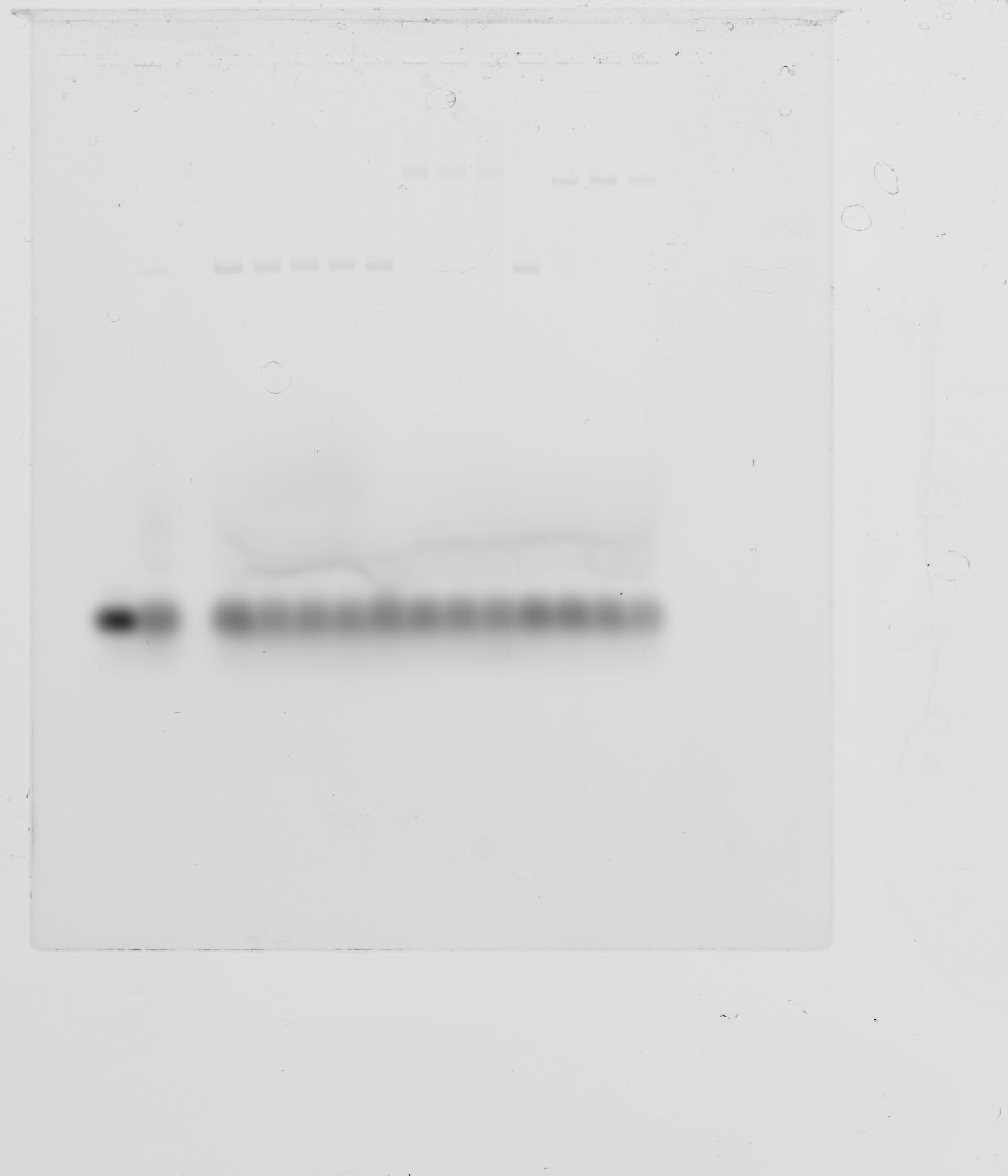

Supplement: Supplemental Information 3 [file peerj-13-19758-s003.zip › Fig1_2_3/Figure 2/Figure 2B/Fig2B.gel]

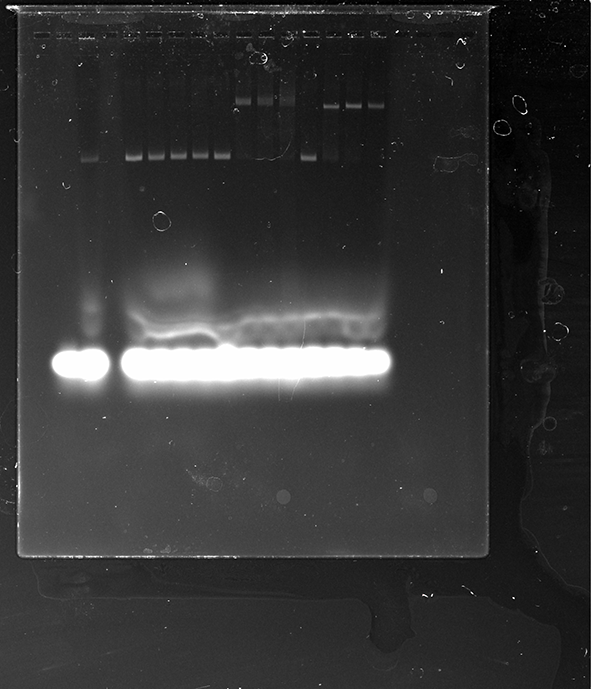

Supplement: Supplemental Information 3 [file peerj-13-19758-s003.zip › Fig1_2_3/Figure 2/Figure 2B/Fig2B.tif]

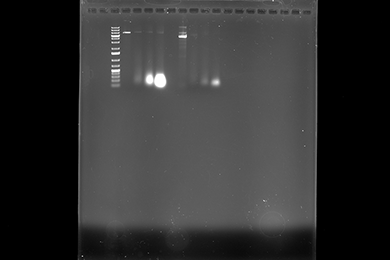

Supplement: Supplemental Information 4 [file peerj-13-19758-s004.zip › Fig4_6/Figure 6/Figure 6a/Fig6_A.tif]

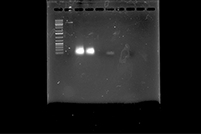

Supplement: Supplemental Information 4 [file peerj-13-19758-s004.zip › Fig4_6/Figure 6/Figure 6b/Fig6B.tif]

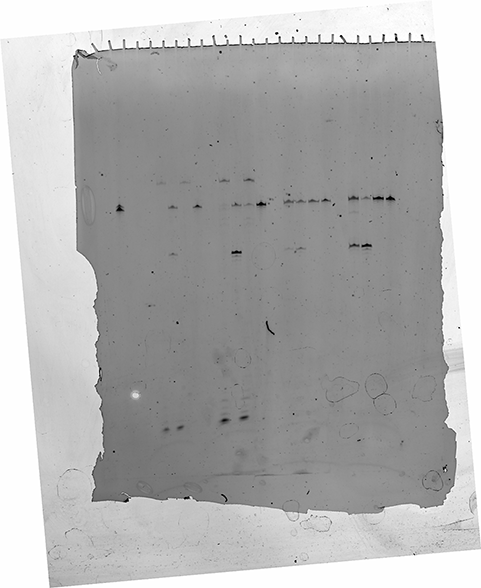

Supplement: Supplemental Information 4 [file peerj-13-19758-s004.zip › Fig4_6/Figure 6/Figure 6c/Fig6C.tif]

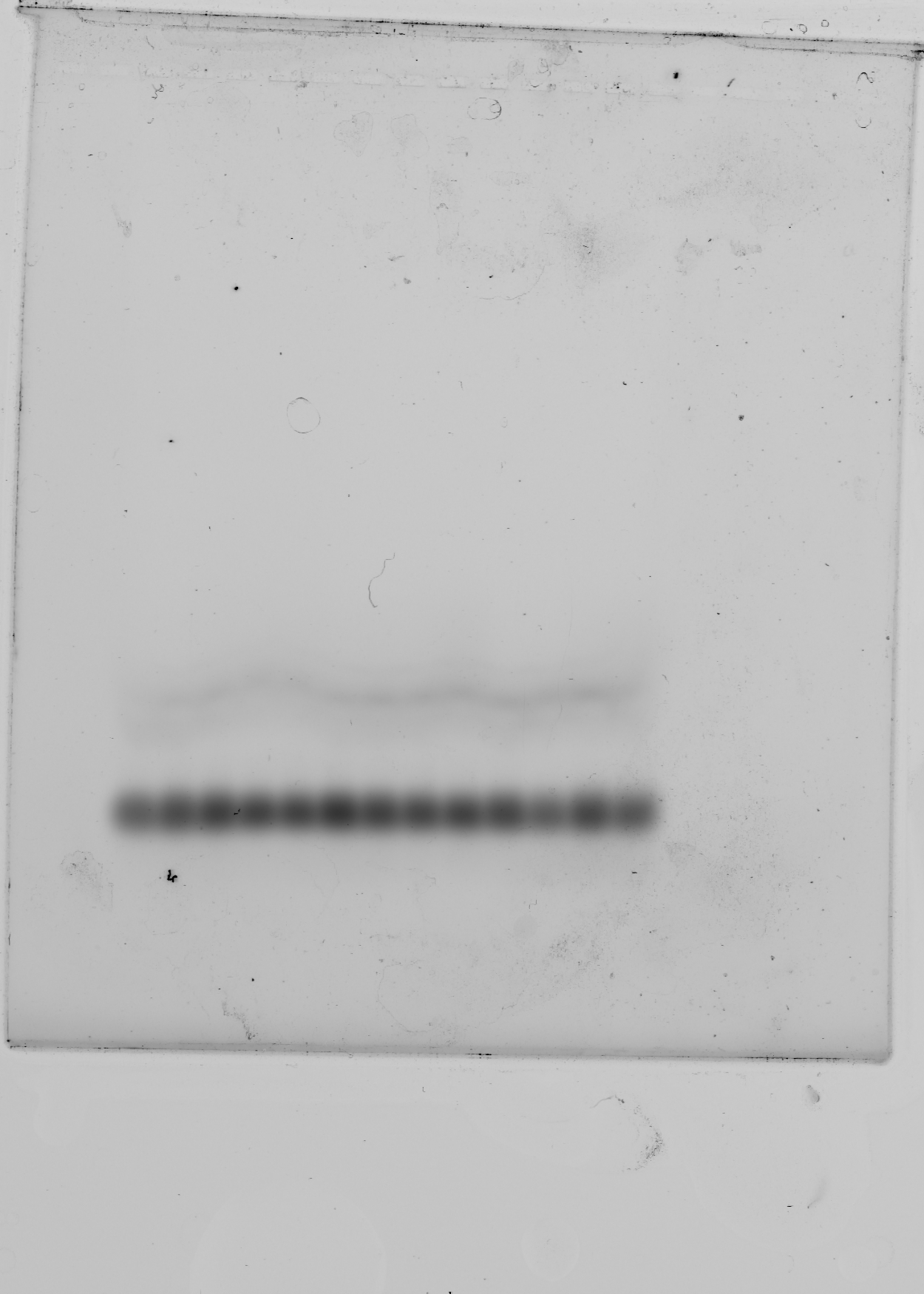

Supplement: Supplemental Information 4 [file peerj-13-19758-s004.zip › Fig4_6/Figure 6/Figure 6c/Fig6C.gel]

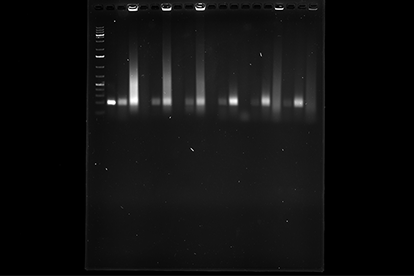

Supplement: Supplemental Information 4 [file peerj-13-19758-s004.zip › Fig4_6/Figure 4/Figure 4A/Fig4A.tif]

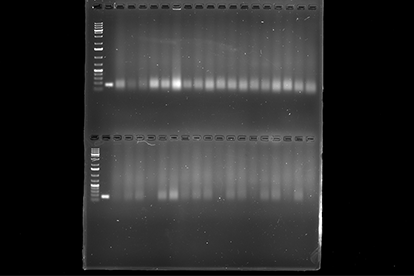

Supplement: Supplemental Information 4 [file peerj-13-19758-s004.zip › Fig4_6/Figure 4/Figure 4B y C/Fig4B_4C.tif]

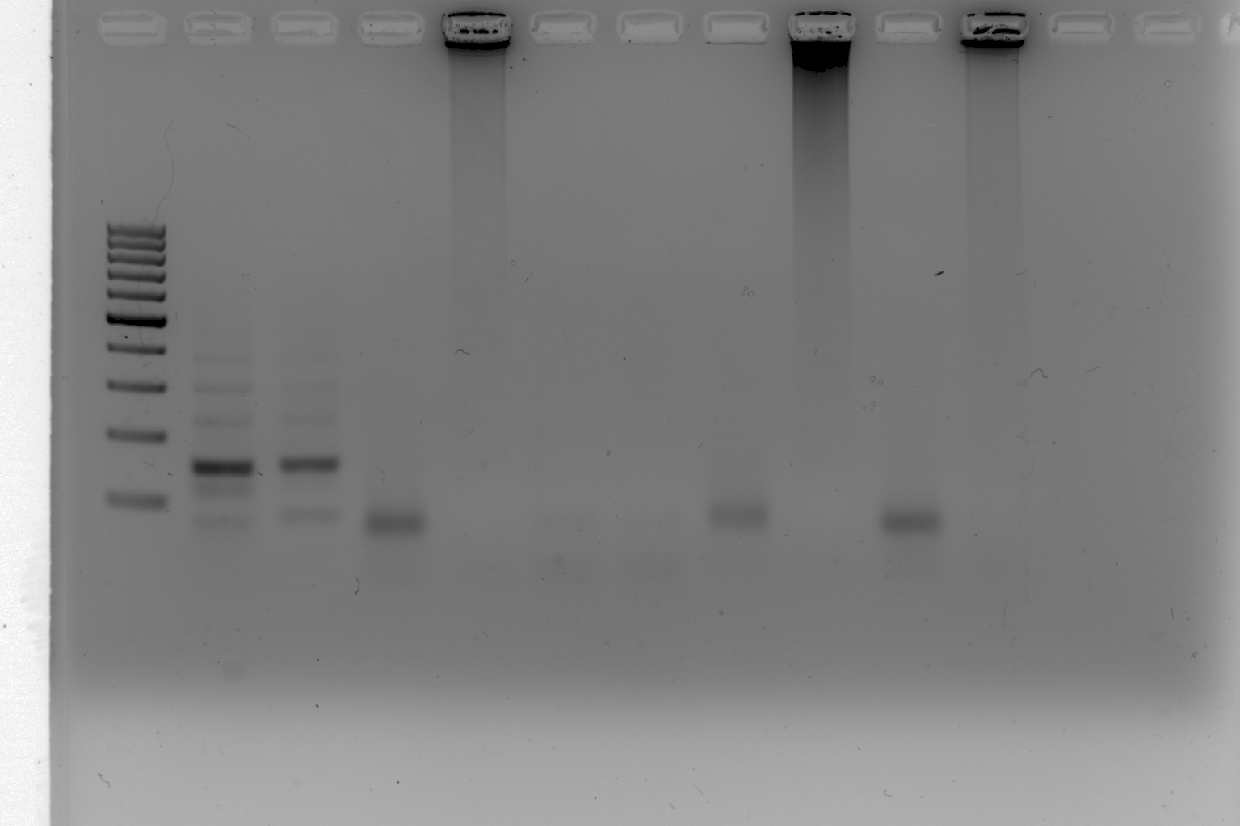

Supplement: Supplemental Information 5 [file peerj-13-19758-s005.zip › Fig5_7/Figure 7/Fig7.jpg]

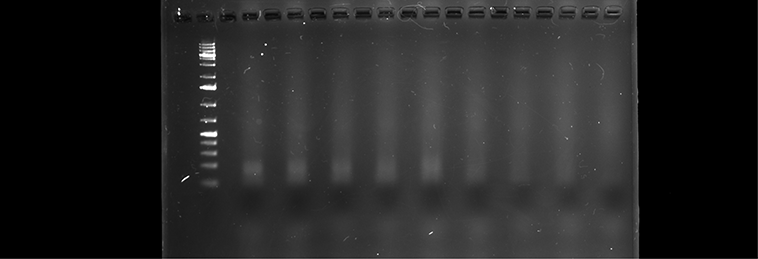

Supplement: Supplemental Information 5 [file peerj-13-19758-s005.zip › Fig5_7/Figure 5/gen n/Fig5_genn.tif]

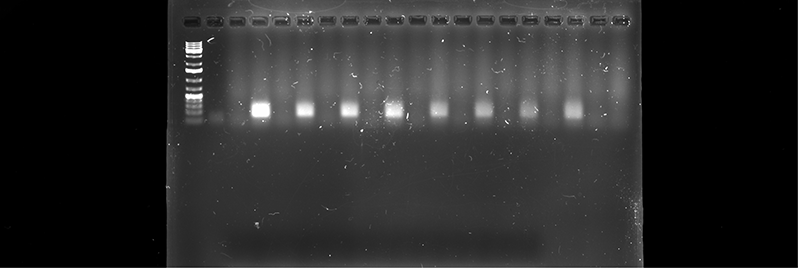

Supplement: Supplemental Information 5 [file peerj-13-19758-s005.zip › Fig5_7/Figure 5/gen p/Fig5_gen_p.tif]

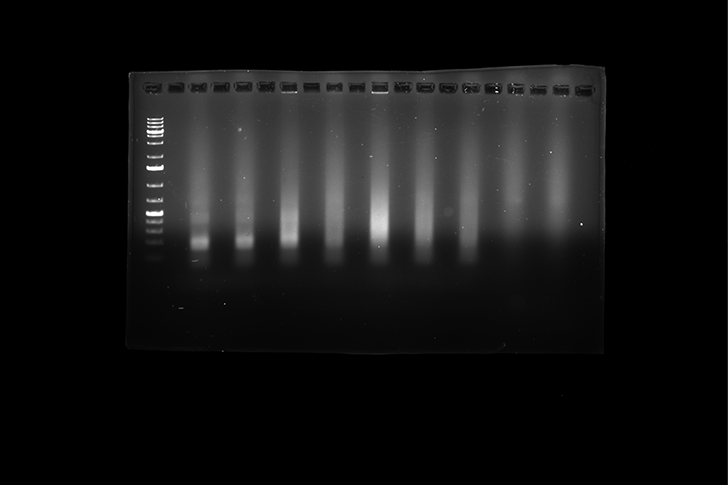

Supplement: Supplemental Information 5 [file peerj-13-19758-s005.zip › Fig5_7/Figure 5/gen e/Fig5_gen e.tif]

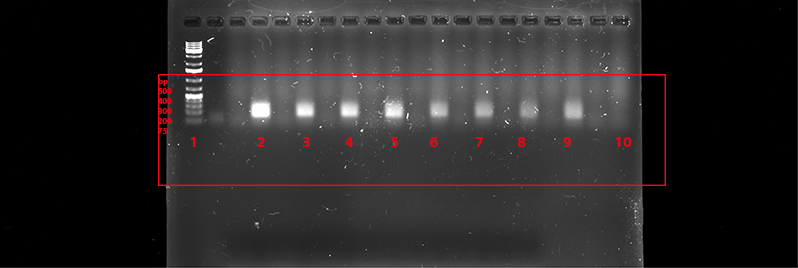

Supplement: Supplemental Information 6 [file peerj-13-19758-s006.zip › Uncroppedgels/Fig5_gen_p_label.tif]

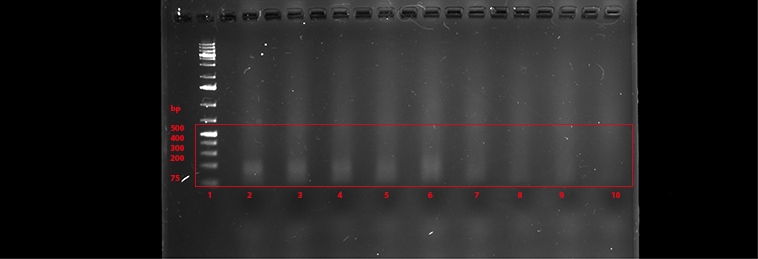

Supplement: Supplemental Information 6 [file peerj-13-19758-s006.zip › Uncroppedgels/Fig5_gen_n_labels.tif]

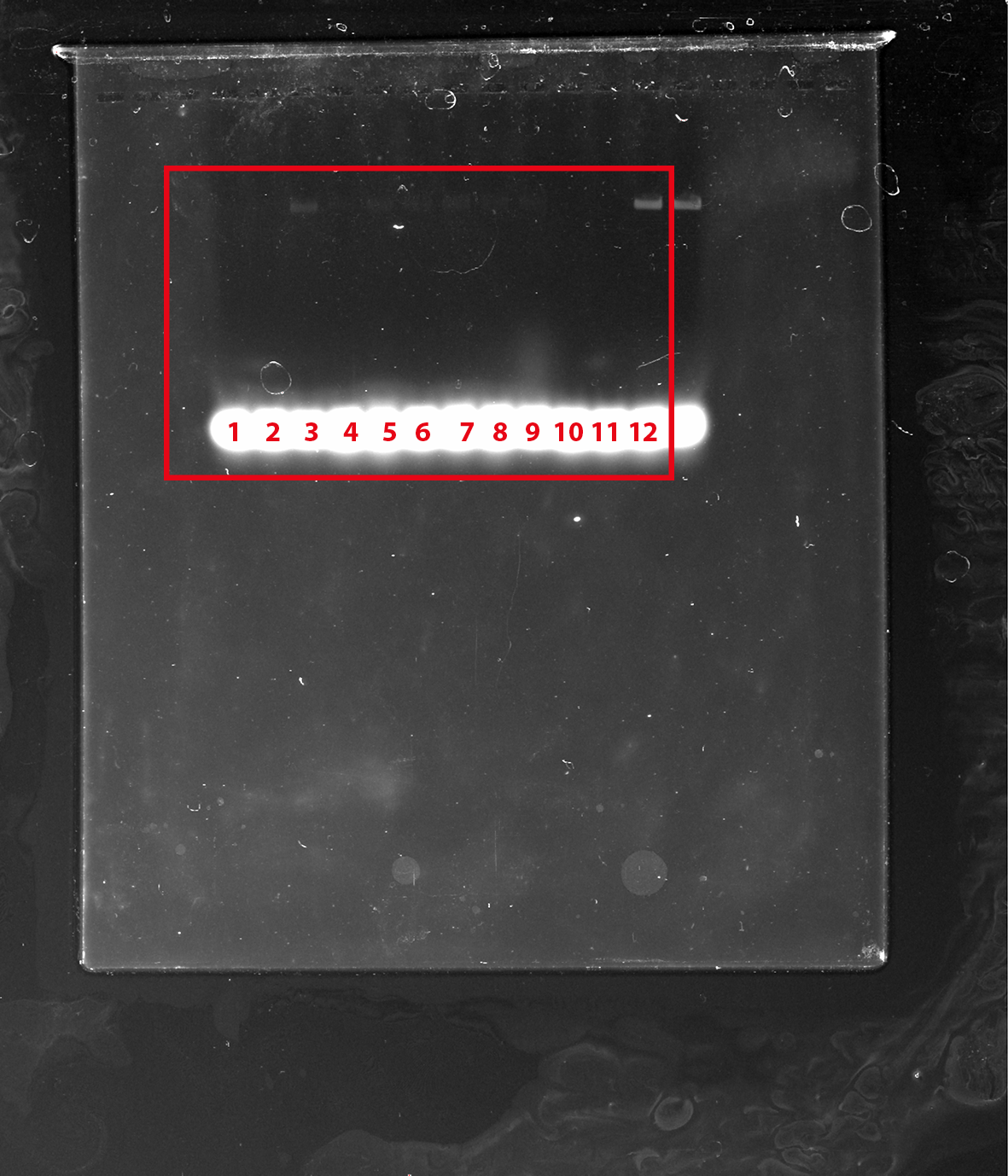

Supplement: Supplemental Information 6 [file peerj-13-19758-s006.zip › Uncroppedgels/Fig1C-labels.tif]

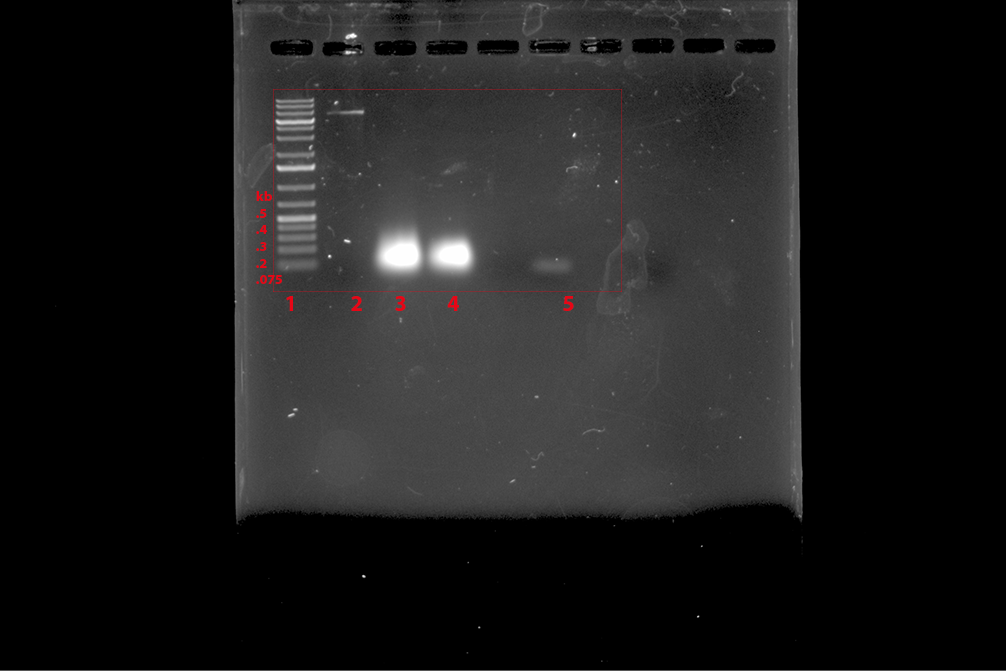

Supplement: Supplemental Information 6 [file peerj-13-19758-s006.zip › Uncroppedgels/fig6B_labels.tif]

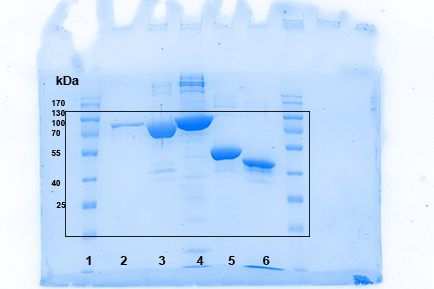

Supplement: Supplemental Information 6 [file peerj-13-19758-s006.zip › Uncroppedgels/Fig2A_labeles.tif]

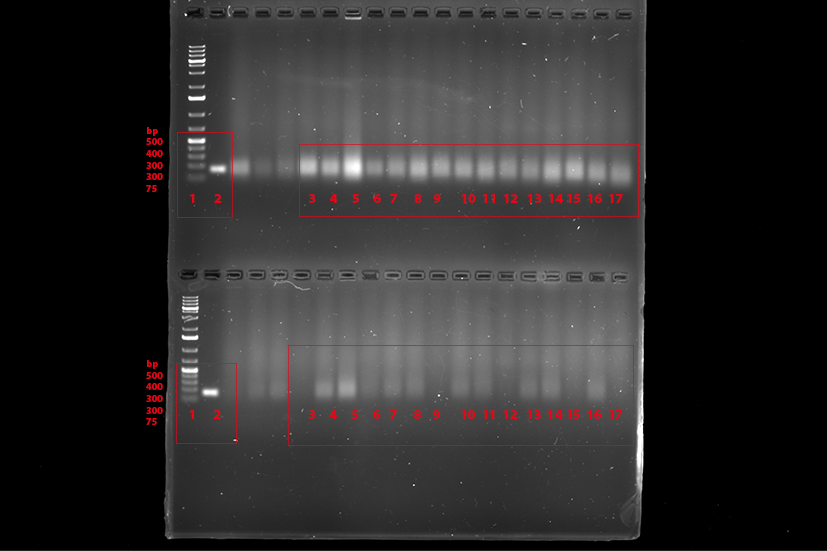

Supplement: Supplemental Information 6 [file peerj-13-19758-s006.zip › Uncroppedgels/Fig4B-4C_labels.tif]

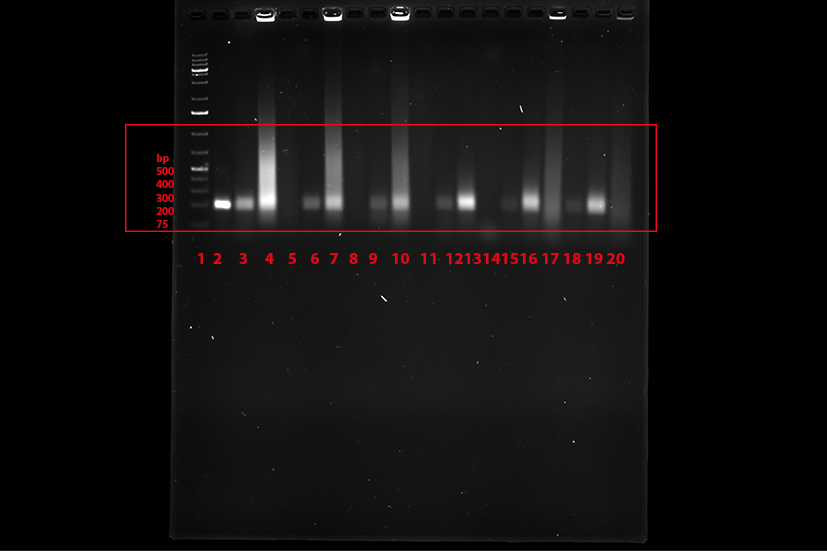

Supplement: Supplemental Information 6 [file peerj-13-19758-s006.zip › Uncroppedgels/Fig4A_labels.tif]

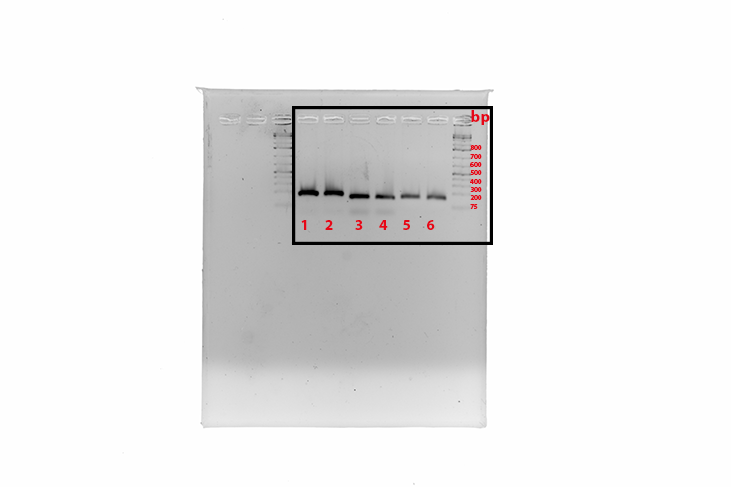

Supplement: Supplemental Information 6 [file peerj-13-19758-s006.zip › Uncroppedgels/Fig3B_labels.tif]

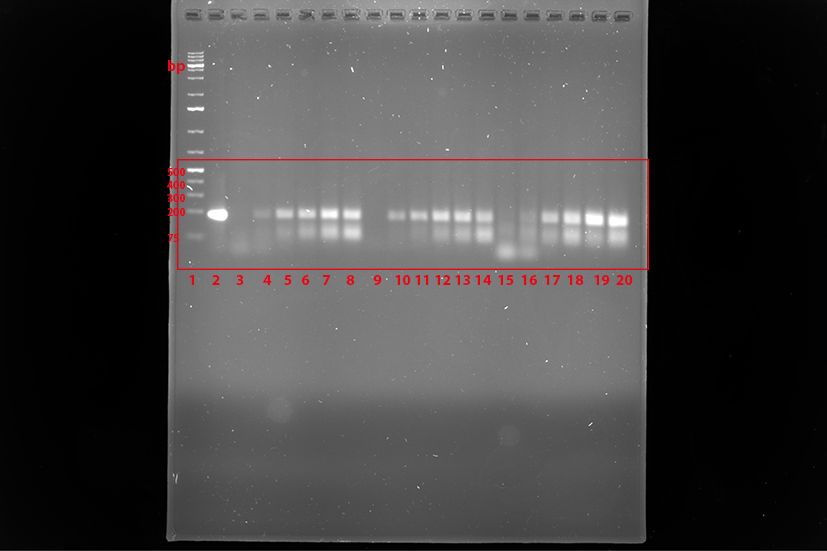

Supplement: Supplemental Information 6 [file peerj-13-19758-s006.zip › Uncroppedgels/Fig3D_labels.tif]

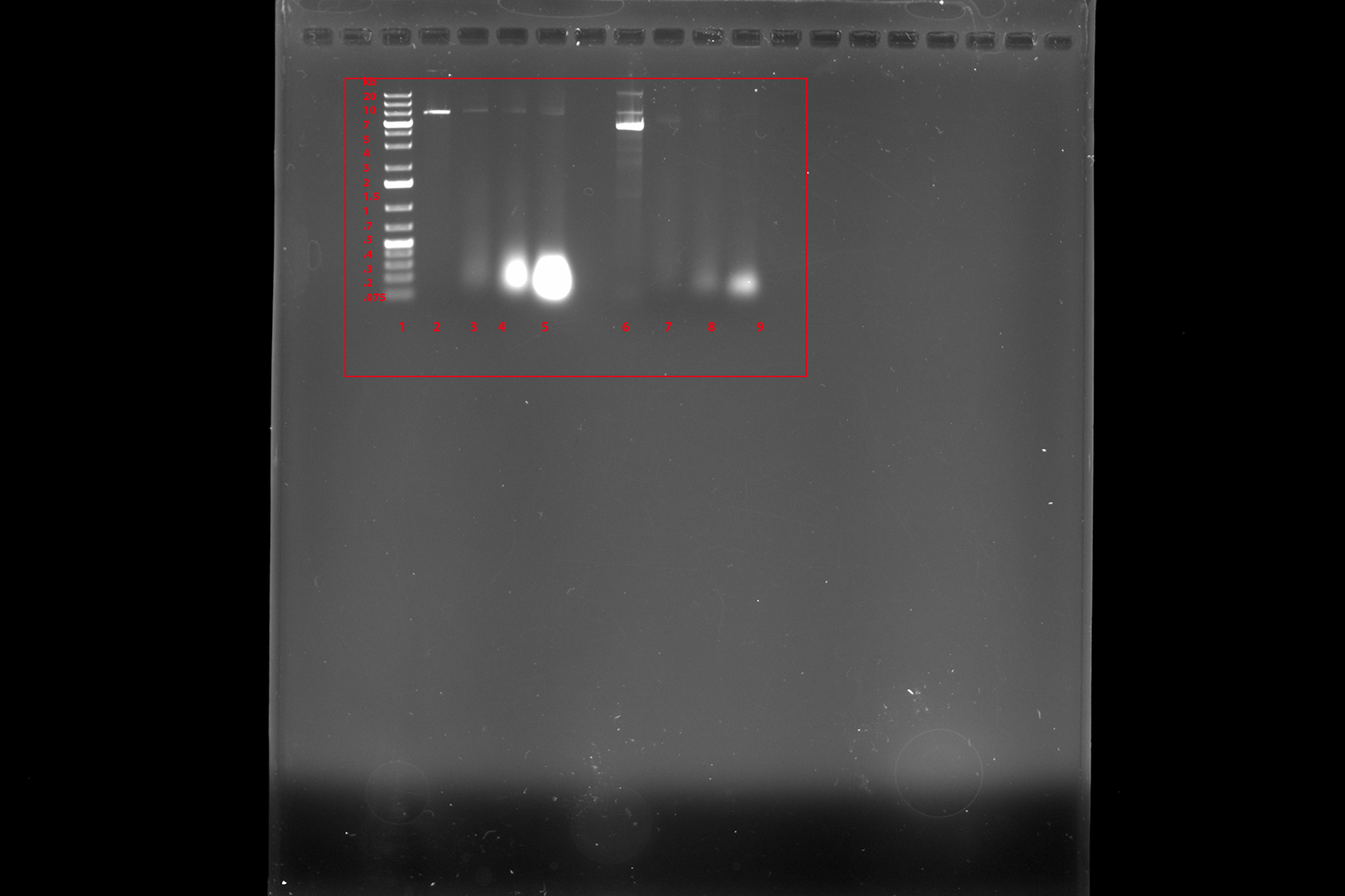

Supplement: Supplemental Information 6 [file peerj-13-19758-s006.zip › Uncroppedgels/Fig6a_labels.tif]

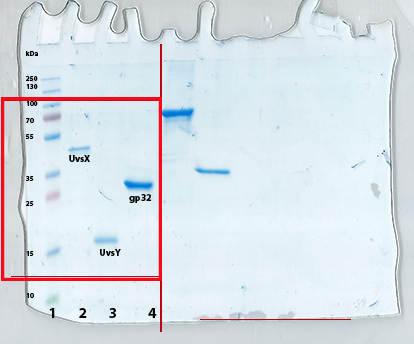

Supplement: Supplemental Information 6 [file peerj-13-19758-s006.zip › Uncroppedgels/Fig1B_labels.tif]

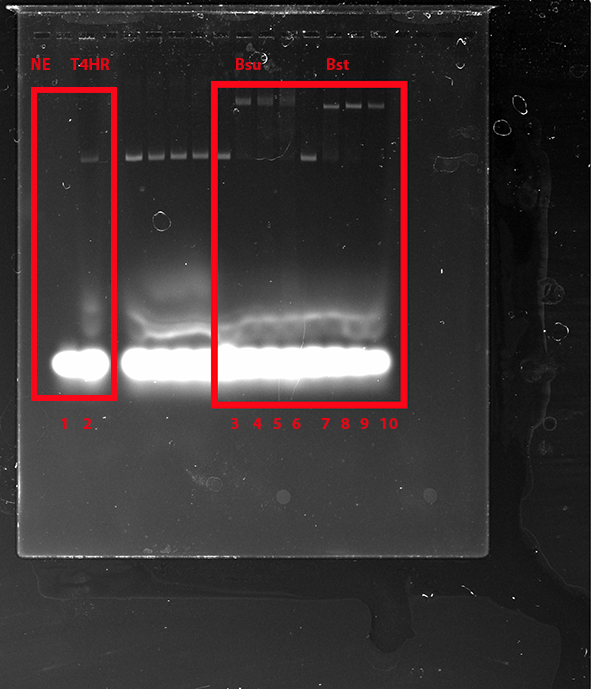

Supplement: Supplemental Information 6 [file peerj-13-19758-s006.zip › Uncroppedgels/Fig2B_labels.tif]

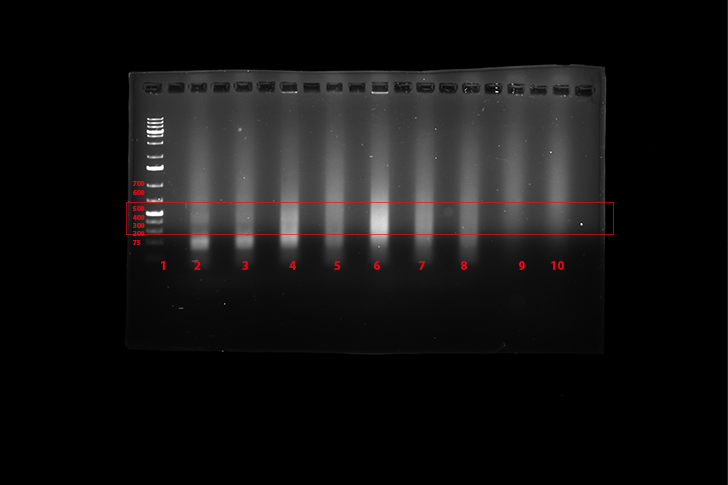

Supplement: Supplemental Information 6 [file peerj-13-19758-s006.zip › Uncroppedgels/Fig5gen_e_labels.tif]

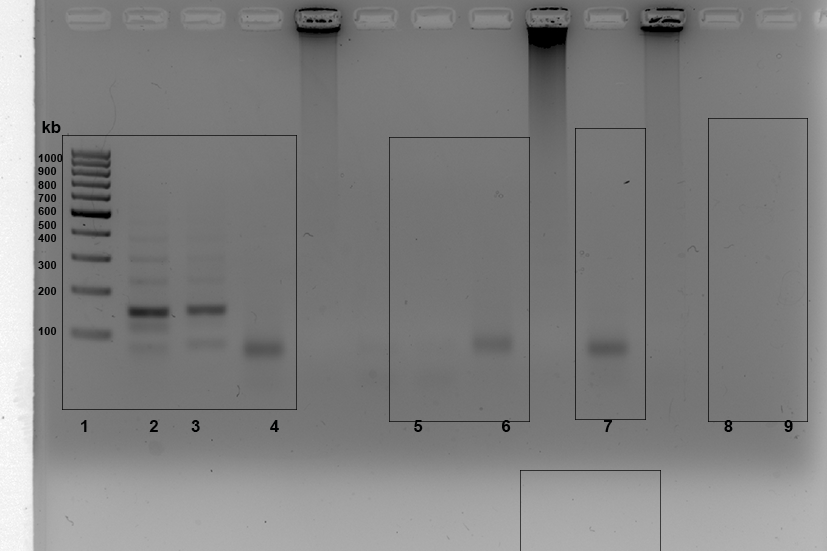

Supplement: Supplemental Information 6 [file peerj-13-19758-s006.zip › Uncroppedgels/Fig7_labels.tif]

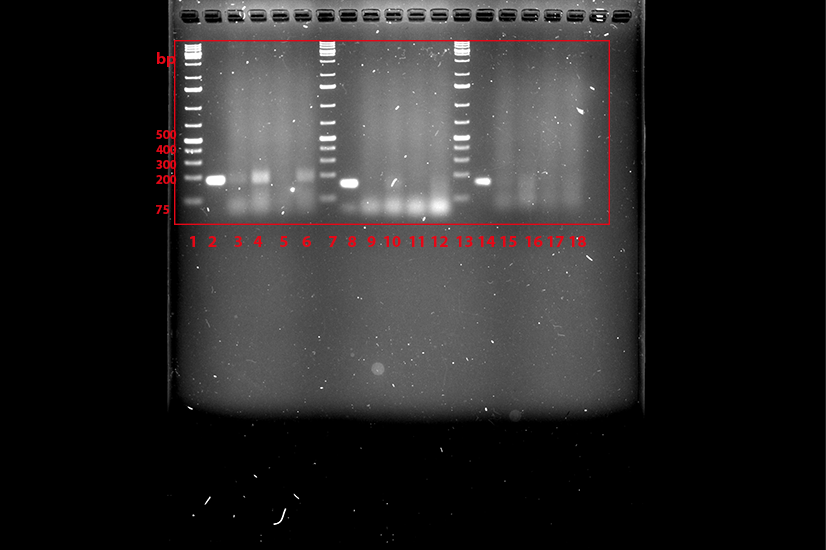

Supplement: Supplemental Information 6 [file peerj-13-19758-s006.zip › Uncroppedgels/Fig3C_labels.tif]

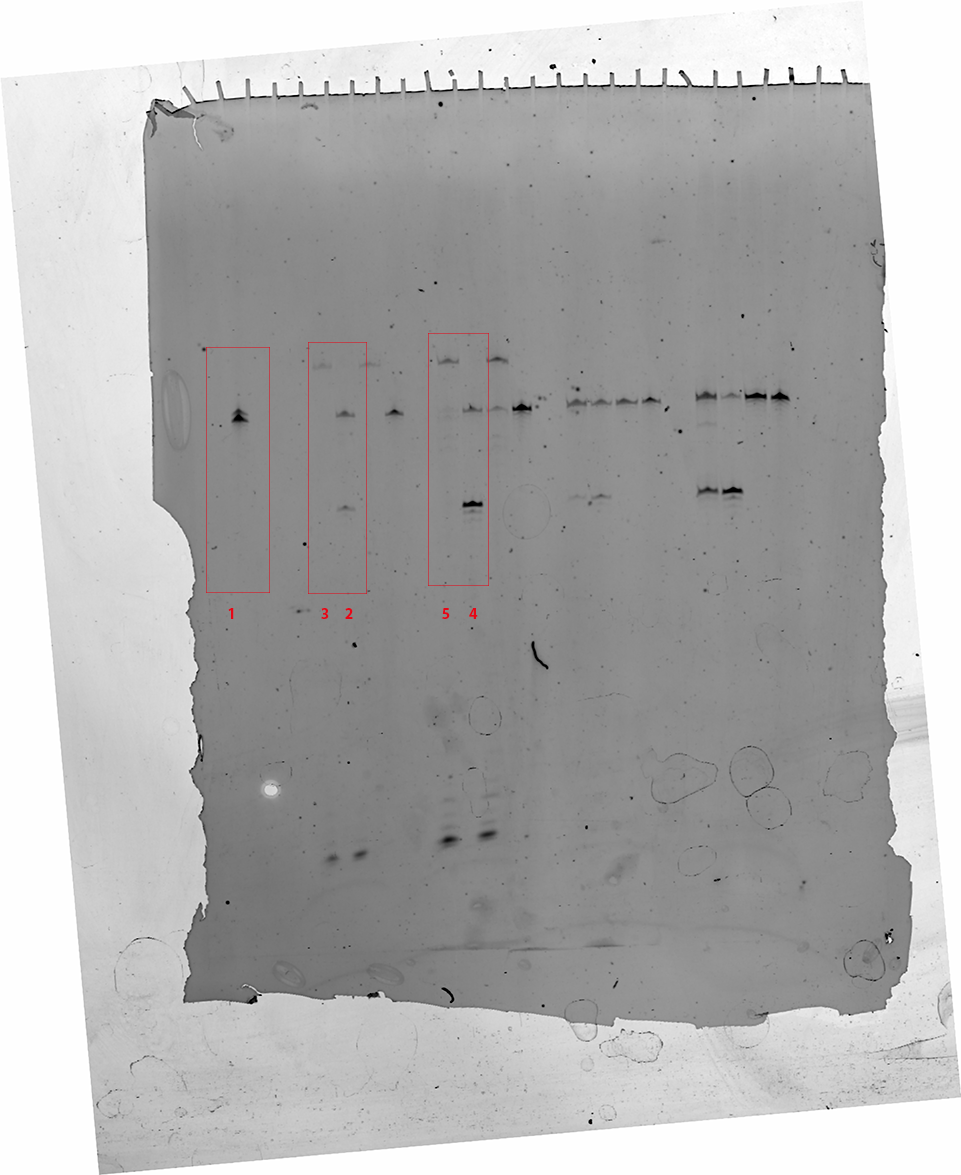

Supplement: Supplemental Information 6 [file peerj-13-19758-s006.zip › Uncroppedgels/fig6C_labels.tif]
